# Exhibits and Exhibitors at the Saratoga Meeting

**Published:** 1902-07

**Authors:** 


### Exhibits and Exhibitors at the Saratoga Meeting.

ONE of the very interesting features of the meetings of the American Medical Association is the display of the manufacturing pharmacists, instrument makers and publishers, generally spoken of as "The Exhibit." It has been growing in significance and value until it has come to be regarded as only second in importance to the meeting itself. The reasons for this are not difficult to discover.

In the first place the exhibitors themselves are among the most influential and successful business men in the country, and not a few of them are men of scientific attainment. They are chemists, pharmacists, physiologists, litterateurs, publishers, manufacturers, and men of affairs in the broadest and best sense of the term. They are doing a most important work supplementary in character to that of the physician. They establish what is virtually a museum of medical and surgical instruments, apparatus and dressings, pharmaceutical preparations, food and drink for the sick and for infants, utensils for chemical, microscopical and biological laboratories, sanitary and hygienic appliances and devices, hospital and office furniture, and of the literature of the medical and allied sciences.

The exhibit hall, or museum as it should be called, is usually located at some place convenient to the building where the meeting is held and easily becomes one of the attractions of convention week. It is a place where old friends may meet and visit in an informal manner and it affords a restful break in the daytime proceedings of the association. In Saratoga the location was a happy one—adjacent to the Hathorn spring house, which latter furnished an appropriate reception hall. The exhibits were all of a high class and were well visited and inspected by interested throngs during all the days of the sessions.

There was, however, one essential feature that merits criticism, because it is a growing evil that must be checked before it becomes ruinous. We refer to the high price charged exhibitors for space. When as much as $135.00 is charged for 60 square feet of surface area, and it cost some exhibitors as high as $400.00 to secure adequate space for four days, it will be readily understood that such rent borders on the exorbitant. This item added to all the other necessary exhibit expenses, such as freight, packing, rent of furniture, and numerous miscellaneous items, brings the total expenditure up to a figure that very few will feel justified in making. We understand the aggregate sum received from the exhibitors at Saratoga was between $7,000 and $8,000, or quite enough to meet the expenses of the local committee of arrangements. It is a matter of observation that it will take but little time if these exorbitant charges are continued, to drive out the exhibitors or compel them to organise for themselves and establish museums independently of the association in the cities wherever it meets.

It seems to us, and we have been observers of the situation for twenty years or more, that it would be an exercise of wisdom on the part of the association to assume control of this matter and reduce it to a business basis. A conference should be had with the exhibitors, their interests should be consulted and they should be accorded privileges at reasonable prices that are consistent with the dignity of both parties. The British Medical Association establishes a medical museum in connection with its meetings, publishes the fact in its programs, announces it from the platform and charges very reasonable prices to the exhibitors. The latter easily become great allies and contribute to the success of meetings if they are properly treated.

The local committee of arrangements should not be allowed to exercise further authority in the premises, and we hope that action will be taken which will lead to a correction of what may soon become a great scandal.

